# Method for Resistivity Measurement of Grainy Carbon and Graphite Materials

**DOI:** 10.3390/ma12040648

**Published:** 2019-02-21

**Authors:** Krzysztof Janerka, Jan Jezierski, Marcin Stawarz, Jan Szajnar

**Affiliations:** Department of Foundry Engineering, Silesian University of Technology, 7 Towarowa, 44-100 Gliwice, Poland; krzysztof.janerka@polsl.pl (K.J.); marcin.stawarz@polsl.pl (M.S.); jan.szajnar@polsl.pl (J.S.)

**Keywords:** resistivity measurement, graphite, carbon, cast iron recarburization

## Abstract

The article presents the issue of electrical resistivity measurement of carbon materials. The device that was developed by the authors is described and is the subject of a Polish patent. The innovative approach of the setup is based on the possibility of measuring the resistivity of grainy (powdered, dusty) materials without having to conduct their preliminary pressing. The material that is to be analyzed is placed inside the chamber made of electrically non-conducting material. The sample is then compacted with pneumatically driven pistons and the compaction force can be controlled by the air pressure. The device as proposed by the authors works at a pressure of 900 kPa, which is equal to the compaction force of 1.2 kN. Resistivity is calculated on the basis of the voltage drop recorded on the sample length. The research covers the analysis of the influence of carbon material grade and compaction force on the resistivity value. It was stated that the resistivity of the graphite materials that were analyzed here changed within the range of 43–172 µm: petroleum coke—360–780 µm; the anthracite—1900–3900 µm. The experimental method presented here can be used whenever carbon materials are present in the form of grains.

## 1. Introduction

The evaluation of any material is mostly conducted in a direct way, e.g., measurement of the geometrical features (length, height, surface quality) or chemical composition analysis. In many cases, this information is sufficient for an assessment of the potential suitability of particular materials for a specific application. For some industrial applications, it is necessary to estimate the physical and electrical properties, specific for given materials group, for example in the case of materials used for carbon and graphite electrodes in metallurgical and foundry industry (in electric arc furnaces). Higher and higher parameters of melting furnaces (higher power supply) are a result of the need for melting processes. It requires the use of better materials for the electrodes with the lowest possible resistivity. The resistivity and high-temperature resistance enable the graphite to be used for refractory materials, batteries and other fuel cells [1]. Graphite is the material ensuring efficient lubrication so, it can be used for electric motors brushes and sealing materials [2]. In such cases, the carbon material is compacted under a high compressive load and the measurements are being made on the ready to use products [3]. Yet very often some technological processes are so complicated that the only base material parameters used to describe their quality do not fully emphasise their properties, e.g. in metallurgical processes, the carburisation process is used to increase the carbon content inside cast iron. It is based on introducing the carbon carrier material (carburiser) with a solid charge or onto the liquid metal surface being melted in the metallurgical furnace. Anthracite, petroleum or pitch coke and synthetic graphite are most often used as carburisers. During the carburisation process, an important parameter is the rate of carbon assimilation by the liquid alloy, i.e. the so-called carburisation efficiency [4,5]. It mostly depends on the carbon content in the carburiser, the ash content and its internal microstructure. The best material is synthetic graphite, then petroleum and pitch coke, and the least favourable material is anthracite. Yet the problem is that materials from all of these groups are similar in their chemical analysis, with carbon content variation only in the range of 1%–2% (98%–99.7% C for the synthetic graphite grades, 97%–99% for petroleum coke and 93%–95% C for anthracite) [6,7,8]. Yet, finally, the rate of carbon assimilation by liquid metal inside each of these groups can vary even by 20%.

One of the parameters which can much better combine carburiser quality with its assimilation rate by liquid metal is electrical resistivity. Electrical resistivity, or simply resistivity, is a feature of the material showing how large material resistance against the electrical current is. Due to the wide and increasing range of graphite applications, especially in composition with other materials for example in the electronics industry, there are many articles describing its electrical conductivity and resistivity. In most cases this applies to high purity materials after specific treatment, leading to achieving the best possible electrical properties. Depending on the level of purity, crystallographic structure order, apparent density, shape and size of the grains and even the particular supplier, the values given by in literature may vary significantly. And so, for selected sources the graphitic material resistivity equals:

- from 7.62 to 30.48 µΩm for apparent density 1560–1880 kg/m^3^ [9],
- from 15.40 to 21.00 µΩm for apparent density 1800 kg/m^3^ [1],
- from 68.68 to 91.43 µΩm for apparent density 1650–1900 kg/m^3^ [10].

The method of resistivity measurement of dense-like rods, sheets, sections made of graphite or carbon materials was presented in the ASTM 619-98 standard [11]. Unfortunately, it does not cover the resistivity of grainy materials that is typical of carburisers. We can obviously compact these materials by pressing them under a pressure of ca. 300 MPa and make them dense enough, but we must use a high load press with special tooling [11,12,13]. Yet the resistivity values recorded for material samples prepared this way differed from those of loose materials. It is necessary to add some kind of binder to compact carburisers, which strongly affects the results. Thus the method presented in this paper, which is based on resistivity measurement for quality assessment of the carburising material supplied to the foundry plant with the grain size it possesses, typically seems to be the best [14]. The solution presented here is the subject of a Polish patent P.411903, titled: ‘Method and set up for carbon material electrical resistivity measurement’ [14]. The issue of resistivity and conductivity of various carbon and graphite materials was and still is the subject of many research studies and analyses [15,16,17,18,19].

## 2. Materials and Methods 

**Figure 1:**
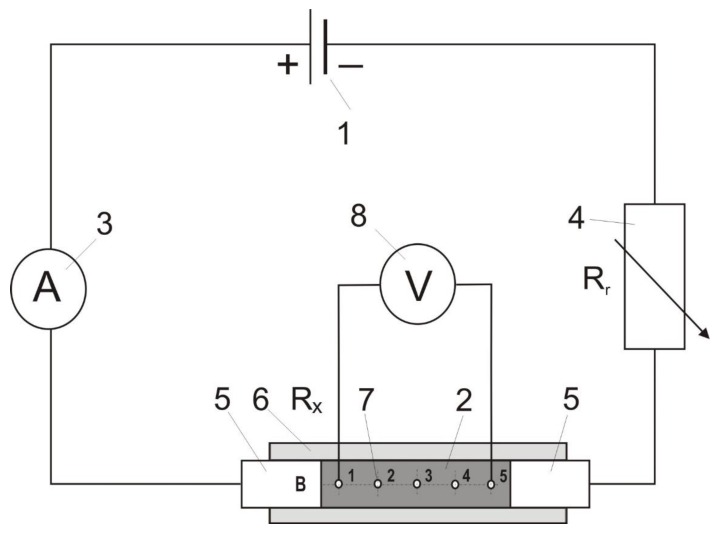
Measuring stand scheme.

There are several methods to conduct resistivity measurement of solid (compact) materials. These are direct measuring methods and indirect ones that are based on resistivity calculation using electric current voltage and its intensity. In the case presented here, we used the algorithm of resistivity estimation on the basis of a voltage drop along the sample [20,21]. Resistivity is denoted by the Greek symbol ρ and is strictly connected to electrical resistance. Electrical resistance R of any conductor which the electrical current is going through depends on its geometrical parameters: (R = ρL/S). In the case presented here, length L is the distance between the measuring electrodes. Electrical resistance R can be calculated after recording voltage U and current intensity I. The resistivity SI unit is 1 Ω·m (ohm meter) [20,21]. The experimental stand is presented in Figure 1.

The recording setup consists of an electric generator (1) with series connection with the carbon material being analysed (2), a resistor of RX, resistance, an ammeter (3) and a rheostat Rr (4), which is necessary for small resistance recording. The resistor RX, situated between the two actuators’ pistons (5), is replaced by the material sample, which is placed in the measuring chamber (6) made of a non-conducting material (plastic). The chamber possesses five measuring holes (7) in the given distances along the two perpendicular planes (A and B). The chamber is 140 mm long, the inner diameter is 12 mm and the holes are made with a distance of 15 mm within one another. The voltage drop is measured when the contact tips of the meter (8) are successively introduced into the measuring holes (7). Ten recordings proceeded (1–2, 1–3, 1–4, 1–5, 2–3, 2–4, 2–5, 3–4, 3–5, 4–5) in the A plane and, analogically, ten recordings were done in the B plane.

The electrical resistivity of various carburisers was measured during the research. There were three materials: synthetic graphite (GS), anthracite (ANT) and petroleum coke (KN). The chemical composition of these materials is provided in Table 1.

The measuring chamber, filled with the analysed material, was situated between the compressing pistons. Thanks to the pressure reducer, the pneumatic actuators’ working pressure was adjusted. The research stand made it possible to adjust the pressure within the range of 0–1.1 MPa. The pressure of the gas supplied from the general compressed air installation was adjusted by means of Festo reducer coupled with the manometer with the range 0–1.6 MPa. The gas pressure on the actuators moving the compressing pistons was precisely checked by another manometer with the working range 0–1 MPa and the accuracy class 0.1. The experiments presented here were carried out under a pressure of 0.9 MPa or in the range of 0.1–0.9 MPa. Then the carburiser was compressed by pistons to which tips were connected to close the circuit. A stabilised power supply was turned on, the voltage was set at 12 V and the rheostat value was adjusted (these settings were set for each recording). The current intensity inside the circuit was measured for such parameters. Then the tips were introduced successively into the measuring holes and the voltage drop inside the analysed material (carburiser) was measured. A total of 20 measurements were made for each material, the necessary calculations were done and the average resistivity value was calculated for them.

## 3. Results and Discussion

**Figure 2:**
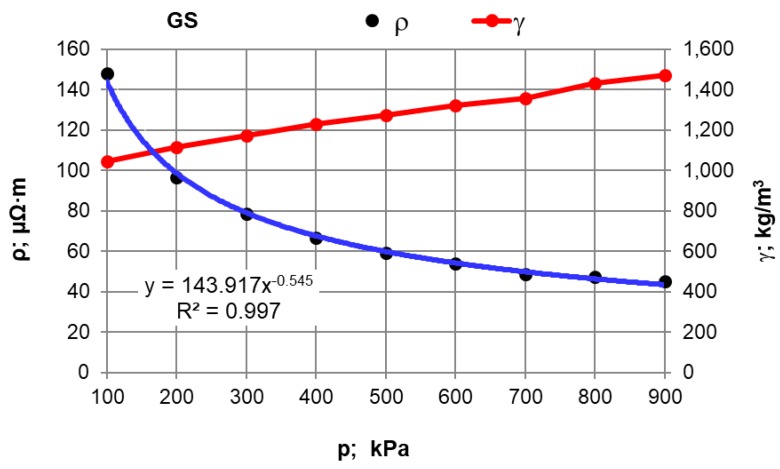
Resistivity and density versus the pressure value for the GS carburiser.

**Figure 3:**
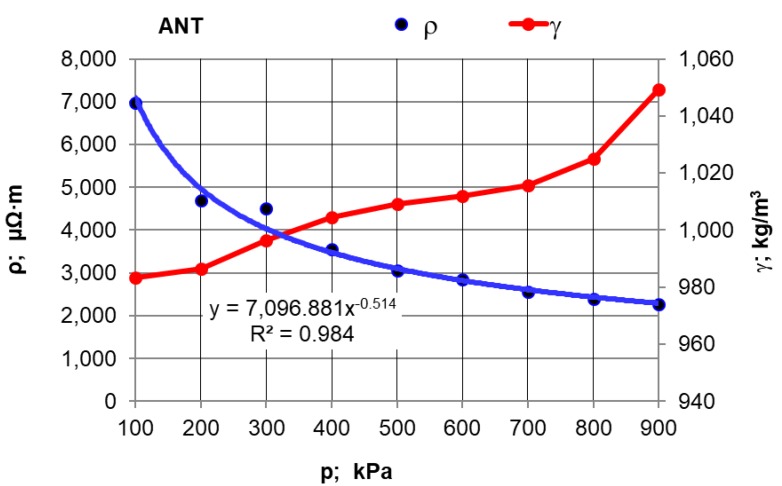
Resistivity and density versus the pressure value for the ANT carburiser.

**Figure 4:**
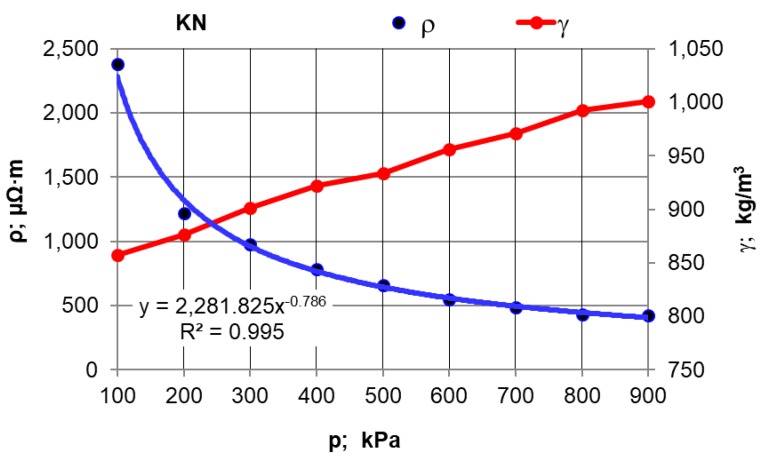
Resistivity and density versus the pressure value for the KN carburiser.

The first stage of the research plan comprised compressive force influence on the electrical resistivity value. Moreover, measurements were made of the effect of pressure compacting the sample material inside the chamber on the density of selected carburisers calculated as γ = m/(S L), where: m—sample weight; kg, S—inner measuring chamber (pipe) surface area; m^2^, L—length of the material compacted inside the chamber; m. The results for synthetic graphite (GS) are presented in Table 2 and in Figure 2, and for the rest of the materials in Figure 3 and Figure 4.

**Figure 5:**
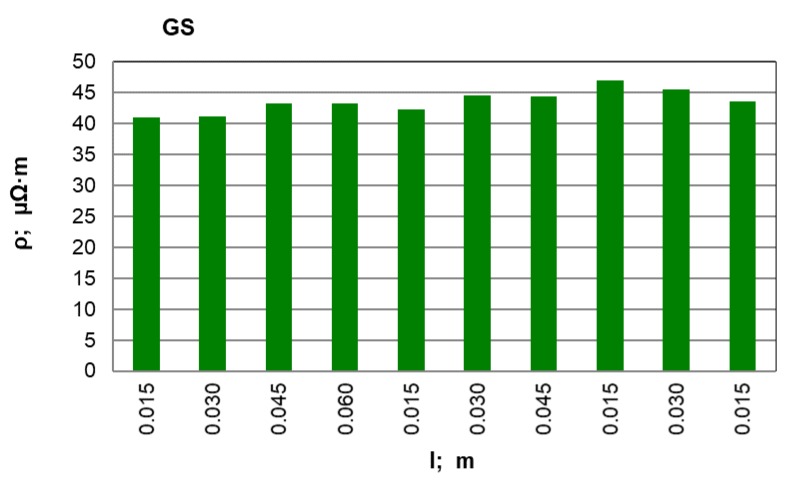
GS material resistivity for various measuring electrode distances l; m.

**Figure 6:**
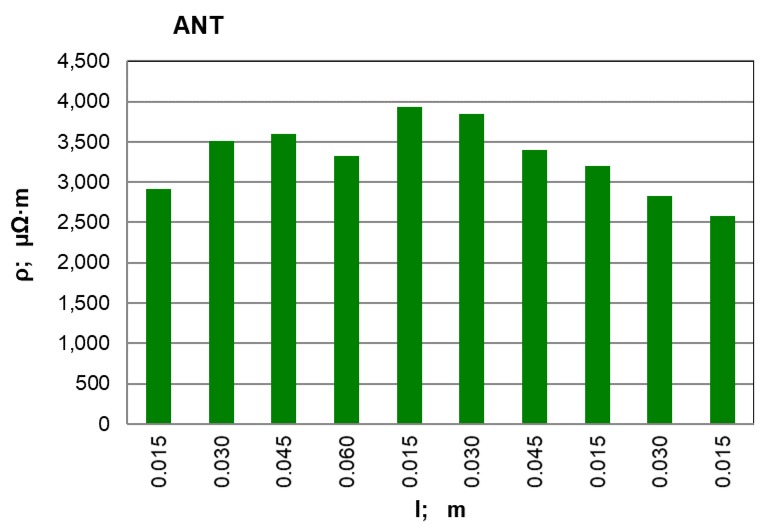
ANT material resistivity for various measuring electrode distances l; m.

**Figure 7:**
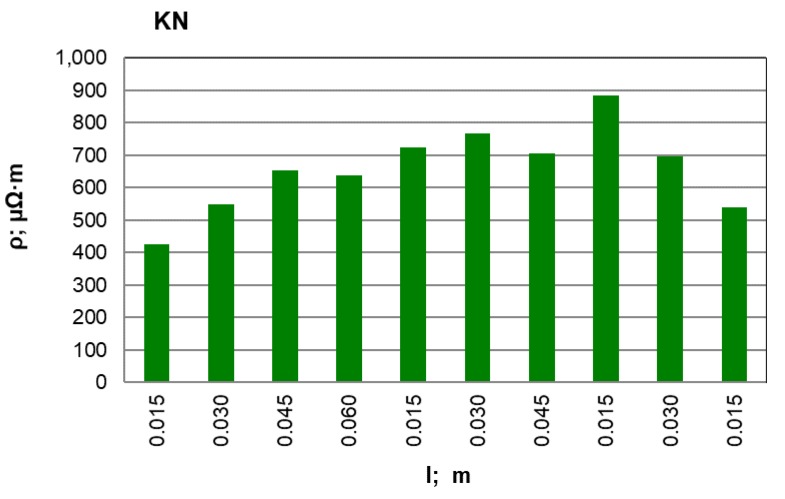
KN material resistivity for various measuring electrode distances l; m.

After conducting an analysis of the recorded and calculated data it can be stated that along with an increase in the force compressing the carbon material inside the measuring chamber, its density obviously increases while the resistivity value significantly decreases. The largest resistivity drop was recorded for the pressure change from 100 kPa to 200 kPa for all materials. It can be observed that inside the pressure range of p = 700–900 kPa, resistivity changes in a relatively small range. This suggests that the pressure values set (900 kPa) for the resistivity measurement of grainy carbon materials are appropriate. For most of the analysed materials, their density increases proportionally to the pressure value. The next experimental stage was measuring resistivity for a pressure of 900 kPa and for various measuring electrode distances for the carbon and graphite materials that had previously been selected. The results of the resistivity measurements and the calculations are presented in Table 3 for graphite type GS1 (as an example) and in Figure 5, Figure 6 and Figure 7 for all materials.

When the above graphs are analysed, it can be stated that the resistivity value is similar along the whole sample length only for synthetic graphite types GS1 and GS3. For the rest of the materials, the resistivity increases in the middle part of the measuring chamber. This results from the non-uniform compression of the material along the whole chamber length; it is particularly visible for anthracite and petroleum coke. To achieve the best resistivity recordings, measurements were made for various electrode distances (0.015, 0.030, 0.045 and 0.060 m) along the whole chamber length. Then the average value of all the recordings was calculated for each material and for the minimum, maximum and standard deviation. For comparison, the resistivity value for the largest measuring electrode distance (l = 0.060 m) is also presented. The results are presented in Table 4.

On the basis of the measurements and the calculations, it can be stated that anthracite (ANT) possesses the largest resistivity among the analysed materials and it is 3409.9 µΩm. The next is petroleum coke (KN), with an average resistivity equal to 660.7 µΩm. Synthetic graphite (GS) has the lowest resistivity equal to 43.10 µΩm. During the average resistivity value analysis for 20 measurements, it can be observed how close it is to the value calculated for the longest measuring electrode distance of 0.060 m. Thus there should be a discussion if it is correct to make some simplifications and to calculate the resistivity on the basis of voltage measurement for the largest possible electrode distance. Yet we must also consider that a significant error can be made because a single recording is always more sensitive than the average value from 20 measurements.

## 4. The Application of the Tested Materials for Cast Iron Recarburization

As it was mentioned previously, one of the areas of carbon materials application is metallurgy, where the recarburization process is carried out to increase the carbon content during the cast iron melting. From the technological point of view, the most important parameters in that process are the efficiency (recarburization effectiveness and carbon assimilation ratio by liquid alloy) and recarburization process rate. These parameters decide of the recarburization process time and the amount of carburizer necessary to introduce to achieve the desired carbon concentration in a liquid melt. The efficiency (recarburization effectiveness is typically given in percentage according to the Equation (1):

(1) $$E=M_{m}\frac{C_{K}-C_{p}}{M_{n}\cdot C_{n}}\cdot 100\%.$$

Recarburization process rate is calculated according to the formula: *S =* *ΔC/t,* where *ΔC* – the carbon growth in liquid alloy in %, *C_p_*—the initial carbon content in %, *C_k_*—final carbon content in %, M_m_—alloy weight in kg, *M_n_*—carburizer weight in kg, *C_n_*—carbon content in the carburizing agent in %, *t*—recarburization process duration time.

### 4.1. Carburizer Introduction with a Solid Charge

The carburizer agent may be added into solid charge composition when electrical induction furnaces, electric arc furnaces or cupolas are used. This method ensures high efficiency without the prolongation of the melting process duration time. Therefore, it makes possible not only the carbon content correction but the so-called synthetic cast iron production (on the base of steel scrap), too. That is why such a method is widely used in cast iron melting. Table 5 presents the results of liquid iron recarburization results when the melting was carried out on the base of the steel scrap only. The experiment−s were conducted in the high-frequency induction furnace of 20 kg capacity. The synthetic graphite (GS), anthracite (ANT) and petroleum coke (KN) were used.

The experiments showed the best carburizer from the metallurgical point of view is the synthetic graphite then petroleum coke and the anthracite. It seems that carbon materials with higher specific resistance are worse carburizers because the recarburization efficiency is lower.

### 4.2. Carburizer Introduction on the Liquid Alloy Surface

This method is the most common one for the electric induction furnaces bot for the synthetic cast iron and this produced on the pig iron base. This is due to the fact that after the solid charge is melted down the sample for chemical analysis is taken and the real carbon deficit in the liquid alloy is estimated. In Table 6 the experimental results with this method of recarburization were presented. The recarburization rate S was given in %.

The recarburization efficiency of the method of carburizer throwing on the liquid alloy surface is much lower than for its addition into solid charge. When we compare the results achieved for the same materials for both methods, the average drop of 15–20% is visible. The highest efficiency and recarburization rate is achieved for the synthetic graphite, petroleum coke and the anthracite, respectively.

## 5. Conclusions

The proposed procedure, as well as the experimental device, makes it possible to estimate the resistivity of grainy carbon and graphite materials. The results allow us to diversify graphite materials of similar chemical composition (GS, ANT, KN). These data are all the more valuable since the preliminary analysis of graphite material resistivity and the rate of carbon assimilation by a molten alloy show that the lower the resistivity is, the higher the carbon assimilation rate is during the carburisation process.

The material characterised by the highest resistivity is anthracite (ANT). Its average resistivity value is at least of an order of magnitude higher than the average value of the rest of the materials. However, synthetic graphite (GS) has the best electrical conductivity. Much higher anthracite resistivity in comparison to graphite may be the result of anthracite’s less ordered crystallographic structure and its higher contamination rate (oxides, mineral inclusions). It may also be that the anthracite grains have higher strength so, even under high pressure (0.9 MPa), they are not packed in measuring chamber in the same way as the synthetic graphite grains.

When the influence of the pressure compressing the material inside the measuring chamber on the resistivity is analysed, it can be stated that the material’s electrical resistivity decreases together with the carbon material compression rate. It can be observed, however, that these changes are small under a pressure of over 700 kPa, which in the authors’ opinion justifies the material sample compressive pressure estimation for 900 kPa.

The cast iron recarburization experiments both with the carburizer into solid charge and on the liquid alloy surface introduction methods showed the best recarburization process indexes (efficiency and recarburization rate) were achieved for the carburizing agent with the lowest specific resistance. 

The main goal of the research presented here was to apply the described method in order to evaluate carburisers that are used in metallurgical processes. Yet we believe that both the measuring procedure and the results obtained can be used in other applications.

## Figures and Tables

**Table 1:** The chemical composition of the carburisers.

Material Grade	C %	S %	Volatile Parts %	Ash %
Synthetic graphite GS	99.20	0.05	0.30	0.60
Synthetic graphite GS2	99.35	0.015	0.08	0.57
Synthetic graphite GS3	99.35	0.02	0.25	0.57
Anthracite ANT	95.75	0.16	0.77	3.55
Petroleum coke KN	99.31	0.82	0.21	0.48

**Table 2:** Example set of resistivity and density values for synthetic graphite GS.

p; kPa	U_V_; mV	I_A_; A	l; m	R; Ω	L; m	ρ; µΩ·m	γ; kg/m^3^
100	4.422	0.225	0.0150	1.965·10^−2^	0.1205	148.2	1046.28
200	2.882	0.225	0.0150	1.281·10^−2^	0.1133	96.6	1112.77
300	2.347	0.225	0.0150	1.043·10^−2^	0.1075	78.6	1173.14
400	1.985	0.225	0.0150	8.822·10^−3^	0.1027	66.5	1227.99
500	1.763	0.225	0.0150	7.836·10^−3^	0.0991	59.1	1272.22
600	1.603	0.225	0.0150	7.124·10^−3^	0.0956	53.7	1319.35
700	1.452	0.225	0.0150	6.453·10^−3^	0.0928	48.7	1358.74
800	1.409	0.225	0.0150	6.262·10^−3^	0.0887	47.2	1429.80
900	1.345	0.225	0.0150	5.978·10^−3^	0.0863	45.1	1469.90

**Table 3:** Results of the recordings and calculations of GS material resistivity.

Point Number	*U_V_*; mV	*I_A_*; A	*l*; m	*R_X_*; Ω	*R*; Ω	*ρ*; Ω·m	*ρ*; µΩ·m
1-2	1.223	0.225	0.0150	5.436·10^−3^	5.43556·10^−3^	4.098·10^−5^	41.0
	1.100	0.225		4.889·10^−3^	4.88889·10^−3^	3.686·10^−5^	36.9
1-3	2.455	0.225	0.0300	1.091·10^−2^	1.09111·10^−3^	4.113·10^−5^	41.1
	2.349	0.225		1.044·10^−2^	1.04400·10^-2^	3.936·10^−5^	39.4
1-4	3.875	0.225	0.0450	1.722·10^−2^	1.72222·10^−2^	4.328·10^−5^	43.3
	3.704	0.225		1.646·10^−2^	1.64622·10^−2^	4.137·10^−5^	41.4
1-5	5.156	0.225	0.0600	2.292·10^−2^	2.29156·10^−2^	4.319·10^−5^	43.2
	5.069	0.225		2.253·10^−2^	2.25289·10^−2^	4.247·10^−5^	42.5
2-3	1.263	0.225	0.0150	5.613·10^−3^	5.61333·10^−3^	4.232·10^−5^	42.3
	1.238	0.225		5.502·10^−3^	5.50222·10^−3^	4.149·10^−5^	41.5
2-4	2.657	0.225	0.0300	1.181·10^−2^	1.18089·10^−2^	4.452·10^−5^	44.5
	2.625	0.225		1.167·10^−2^	1.16667·10^−2^	4.398·10^−5^	44.0
2-5	3.968	0.225	0.0450	1.764·10^−2^	1.76356·10^−2^	4.432·10^−5^	44.3
	3.957	0.225		1.759·10^−2^	1.75867·10^−2^	4.420·10^−5^	44.2
3-4	1.400	0.225	0.0150	6.222·10^−3^	6.22222·10^−3^	4.691·10^−5^	46.9
	1.352	0.225		6.009·10^−3^	6.00889·10^−3^	4.531·10^−5^	45.3
3-5	2.721	0.225	0.0300	1.209·10^−2^	1.20933·10^−2^	4.559·10^−5^	45.6
	2.707	0.225		1.203·10^−2^	1.20311·10^−2^	4.536·10^−5^	45.4
4-5	1.299	0.225	0.0150	5.773·10^−2^	5.77333·10^−3^	4.353·10^−5^	43.5
	1.357	0.225		6.031·10^−3^	6.03111·10^−3^	4.547·10^−5^	45.5

**Table 4:** Average resistivity value of the analysed materials.

Material	GS	ANT	KN
Average resistivity	43.10	3409.90	660.70
Resistivity for l = 0.060 m	43.20	3319.80	637.50
Minimum resistivity	36.86	2474.34	408.73
Maximum resistivity	46.91	4654.06	925.76
Standard deviation	2.41	566.28	135.69

**Table 5:** The results of metal bath recarburization by the addition of carburizer to solid charge.

Material	M_m_	M_n_	C_p_	C_k_	ΔC	E
Synthetic graphite GS	12.31	0.255	1.22	3.24	2.03	98.51
Anthracite ANT	12.99	0.255	0.86	2.48	1.56	81.82
Petroleum coke KN	12.67	0.255	1.18	2.96	1.78	89.30

**Table 6:** The results of recarburization by the carburizer introduction on the liquid metal surface.

Material	M_m_	M_n_	C_p_	C_k_	ΔC	E	S
Synthetic graphite GS	12.92	0.085	0.751	1.35	0.599	91.87	4.12
Anthracite ANT	12.91	0.085	0.715	1.09	0.375	59.95	1.61
Petroleum coke KN	12.91	0.085	0.744	1.29	0.546	84.62	2.54
